# Low high-density lipoprotein level is correlated with the severity of COVID-19 patients: an observational study

**DOI:** 10.1186/s12944-020-01382-9

**Published:** 2020-09-07

**Authors:** Guyi Wang, Quan Zhang, Xianmei Zhao, Haiyun Dong, Chenfang Wu, Fang Wu, Bo Yu, Jianlei Lv, Siye Zhang, Guobao Wu, Shangjie Wu, Xiaolei Wang, Ying Wu, Yanjun Zhong

**Affiliations:** 1 Department of Critical Care Medicine, the Second Xiangya Hospital, Central South University, Changsha, 410011 China; 2 Department of Critical Care Medicine, the First Hospital of Changsha, Changsha, 410011 China; 3 Department of Physical Examination Center, the Second Xiangya Hospital, Central South University, Changsha, 410011 China; 4 Department of Oncology, the Second Xiangya Hospital, Central South University, Changsha, 410011 Hunan China; 5 Department of Respiratory, the Second Xiangya Hospital, Central South University, Changsha, China

**Keywords:** COVID-19, Severe acute respiratory syndrome coronavirus 2, High-density lipoprotein cholesterol, Adult, Lipoproteins, Prognosis

## Abstract

**Background:**

The purpose of the study is to describe the blood lipid levels of patients diagnosed with coronavirus disease 2019 (COVID-19) and to analyze the correlation between blood lipid levels and the prognosis of COVID-19 patients.

**Methods:**

In the clinical retrospective analysis, a total of 228 adults infected with COVID-19 were enrolled between January 17, 2020 and March 14, 2020, in Changsha, China. One thousand one hundred and forty healthy participants with matched age and gender were used as control. Median with interquartile range and Mann-Whitney test were adopted to describe and analyze clinical data. The Kaplan-Meier (KM) curve and Cox regression analysis were used to analyze the correlation between high-density lipoprotein cholesterol (HDL-C) and the severity of COVID-19.

**Results:**

Compared with control, COVID-19 patients showed significantly lower levels of total cholesterol (TC) [median, 3.76 vs 4.65 mmol/L, *P* = 0.031], triglyceride [median, 1.08 vs 1.21 mmol/L, *P* <  0.001], low-density lipoprotein cholesterol (LDL-C) [median, 2.63 vs 2.83 mmol/L, *P* <  0.001], and HDL-C [median, 0.78 vs 1.37 mmol/L, *P* <  0.001], while compared with non-severe patients, severe COVID-19 patients only presented lower levels of HDL-C [median, 0.69 vs 0.79 mmol/L, *P* = 0.032]. In comparison with patients with high HDL-C, patients with low HDL-C showed a higher proportion of male (69.57% vs 45.60%, *P* = 0.004), higher levels of C-reactive protein (CRP) (median, 27.83 vs 12.56 mg/L, *P* <  0.001) and higher proportion of severe events (36.96% vs 14.84%, *P* = 0.001). Moreover, patients with low HDL-C at admission showed a higher risk of developing severe events compared with those with high HDL-C (Log Rank *P* = 0.009). After adjusting for age, gender and underlying diseases, they still had elevated possibility of developing severe cases than those with high HDL-C (HR 2.827, 95% CI 1.190–6.714, *P* = 0.019).

**Conclusions:**

HDL-C level was lower in COVID-19 adult patients, and low HDL-C in COVID-19 patients was correlated with a higher risk of developing severe events.

## Background

Coronavirus disease 2019 (COVID-19), caused by severe acute respiratory syndrome coronavirus 2 (SARS-CoV-2), which was first reported in Wuhan in late 2019 [1–5], has spread globally [6–8]. As of August 28, 2020, SARS-CoV-2 has infected more than 24 million people, and caused nearly 828 thousand deaths worldwide [9]. Although we have a certain understanding of the disease characteristics, the effects of the virus on function and metabolism of human beings are yet to be fully known.

SARS-CoV-2 was believed to affect lipid metabolism [10, 11]. Low-density lipoprotein cholesterol (LDL-C) and total cholesterol (TC) levels significantly decreased in COVID-19 patients, while the changes and effects of high-density lipoprotein cholesterol (HDL-C) in COVID-19 were still rarely reported [12–14]. High-density lipoproteins (HDLs) are a family of particles characterized by their ability to transport cholesterol from extrahepatic tissues to liver for metabolism. Although the primary role of HDLs is anti-atherosclerosis [15], many recent studies have found other multiple characteristics of HDLs, including anti-infection, anti-inflammatory, anti-apoptotic or antioxidant functions [16–18]. Moreover, HDLs is believed to play a protective role in many infectious diseases. It was observed that patients with low levels of HDLs showed an increased risk of infection [19–21] and a worse outcome [22–24]. Therefore, it was assumed that HDL levels may be associated with the prognosis of COVID-19 patients. In this study, the clinical characteristics of adult COVID-19 patients with different HDL-C levels were presented and the association between HDL-C levels and the risk for developing severe events were expounded.

## Methods

### Study design

All adult COVID-19 patients confirmed by reverse transcription-polymerase chain reaction (RT-PCR) admitted to Public Health Treatment Center of Changsha, China, from January 17 to March 14, 2020, who were tested for blood lipid levels were enrolled in the present study. Patients who were below 18 years and those who had not been tested for blood lipids were excluded. A total of 1140 control patients were selected from the Physical Examination Center of the Second Xiangya Hospital, Central South University, China from May 1 to June 8, 2020, with matched age and gender at a ratio of 1 to 5. This study was designed according to the STROBE checklist and was approved by the institutional ethics board of the Second Xiangya Hospital of Central South University (No. 2020001).

### Data collection

Two researchers from our team individually reviewed the medical records of included cases. Demographic data, underlying diseases, symptoms throughout the course of the disease, results of first laboratory examination and lung computed tomographic (CT) scans after admission, length of hospitalization, virus shedding time, severity of illness and mortality were all recorded. Lipid and other laboratory tests were finished within 24 h after admission, but lipid levels before onset and during the recovery period were unknown.

### Definition and study endpoints

With reference to relevant guidelines, the criteria for severe cases were developed [25] and they were consistent with our previous studies [26]. Specifically, the following criteria were utilized to diagnose severe events of COVID-19: (1) oxygen saturation < 93%; (2) PaO2/FiO2 ≤ 300 mmHg; (3) respiratory rate ≥ 30 /min; (4) receiving mechanical ventilation; (5) progression of pulmonary lesions > 50% in chest CT within 24 to 48 h; (6) shock; and (7) admitting to intensive care unit (ICU) [25]. SARS-CoV-2 RT-PCR was performed consecutively twice or more after disease remission with a sampling time interval of more than 24 h, and two consecutive negative results were considered virus free [25]; virus shedding time is defined as the time between disease onset (diagnosis date for asymptomatic cases) and the first negative samples without any positive result thereafter. Low HDL-C is defined as below 0.65 mmol/L (25 mg/dl), while high HDL-C is defined as above or equal to 0.65 mmol/L based on previous studies [23, 27]. The primary endpoint was the severity of COVID-19, and the secondary endpoints were mortality, virus shedding duration, and the length of hospitalization.

### Serum lipids measurement

Levels of TC and triglyceride were determined using COP-CE-PAP and GPO-PAP assays, respectively (Hunan Yonghe-Sun biotechnology Co. Ltd., China). The levels of LDL-C and HDL-C were both measured by catalase scavenging assays (Ningbo Ruiyuan Biotechnology Co., Ltd., China). The inter- and intra-assay coefficients of variation for all parameters were < 3.1% and < 3.0%, respectively.

### Statistical analysis

All continuous variables were depicted using Median with interquartile range, and Mann-Whitney test was used to analyze all continuous variables because of their non-normal distributions. The χ2 test or Fisher’s exact test was used to analyze the categorical variables. The Kaplan-Meier (KM) curve with Log Rank test were performed to estimate the cumulative proportion of severe events in non-severe patients after admission according to the HDL-C level. Finally, the effect of low HDL-C level on the risk of severe event risk was estimated using Cox regression model adjusted for the sex, age, and underlying diseases. All analyses were carried out using IBM SPSS version 26 software.

## Results

All 228 adult patients diagnosed with COVID-19 by March 14, 2020 and tested for blood lipid levels were included in this study. Compared with control, COVID-19 patients showed significantly lower levels of TC [median, 3.76 vs 4.65 mmol/L, *P* = 0.031], triglyceride [median, 1.08 vs 1.21 mmol/L, *P* <  0.001], LDL-C [median, 2.63 vs 2.83 mmol/L, *P* <  0.001], and HDL-C [median, 0.78 vs 1.37 mmol/L, *P* <  0.001] (Table 1). Compared with non-severe patients, severe COVID-19 patients only presented lower levels of HDL-C [median, 0.69 vs 0.79 mmol/L, *P* = 0.032] (Table 2), which suggested that low HDL-C may be correlated with severity of COVID-19 patients. Therefore, the effect of HDL-C level on COVID-19 was further analyzed.

**Table 1:** Blood lipid levels of adult COVID-19 patients and control

	Normal range	Control (*n* = 1140)	All patients (*n* = 228)	*P* value
Age, median (IQR), y		45.5 (36.0–60.8)	45.5 (36.0–60.8)	1.000
Sex (male/female)		575/565	115/113	1.000
Total cholesterol, median (IQR), mmol/L	2.33–5.69	4.65 (3.92, 5.49)	3.76 (3.22, 4.26)	**0.031**
Triglyceride, median (IQR), mmol/L	0.25–1.71	1.21 (0.93, 1.36)	1.08 (0.78, 1.44)	**< 0.001**
HDL-C, median (IQR), mmol/L	0.90–1.94	1.37 (1.22, 1.51)	0.78 (0.66, 0.97)	**< 0.001**
LDL-C, median (IQR), mmol/L	0.60–4.14	2.83 (2.27, 3.39)	2.63 (2.21, 3.09)	**< 0.001**

*P* < 0.05 was considered statistically significant (marked in bold)

*COVID-19* Coronavirus disease 2019; *HDL-C* High-density lipoprotein cholesterol; *LDL-C* Low-density lipoprotein cholesterol; *IQR* Inter-quartile range

**Table 2:** Blood lipid levels of adult severe and non-severe COVID-19 patients

	Normal range	Severe (*n* = 44)	Non-severe (*n* = 184)	*P* value
Total cholesterol, median (IQR), mmol/L	2.33–5.69	3.63 (3.04–4.15)	3.81 (3.24–4.34)	0.082
Triglyceride, median (IQR), mmol/L	0.25–1.71	1.08 (0.76–1.36)	1.09 (0.79–1.47)	0.382
HDL-C, median (IQR), mmol/L	0.90–1.94	0.69 (0.59–0.95)	0.79 (0.69–0.97)	**0.032**
LDL-C, median (IQR), mmol/L	0.60–4.14	2.60 (2.19–2.95)	2.65 (2.22–3.10)	0.233

*P* < 0.05 was considered statistically significant (marked in bold)

*COVID-19* Coronavirus disease 2019; *HDL-C* High-density lipoprotein cholesterol; *LDL-C* Low-density lipoprotein cholesterol; *IQR* Inter-quartile range

Clinical characteristics of adult patients with COVID-19, and with different levels of HDL-C were summarized in Table 3 and Table 4. There were no obvious differences in age and underlying diseases between patients with high and low levels of HDL-C. Compared with patients with high HDL-C, those with low HDL-C showed higher proportion of male (69.57% vs 45.60%, *P* = 0.004), lower proportion of headache (2.17% vs 16.48%, *P* = 0.011) and nausea (4.35% vs 15.38%, *P* = 0.048) (Table 3), as well as higher levels of C-reactive protein (CRP) (median, 27.83 vs 12.56 mg/L, *P* <  0.001) (Table 4).

**Table 3:** Baseline Characteristics of COVID-19 patients with different levels of HDL-C

	Low HDL-C (*n* = 46)	High HDL-C (*n* = 182)	*P* value
Sex (male/female)	32/14	83/99	**0.004**
Age, median (IQR), y	46.0 (37.8, 59.0)	45.0 (35.0, 61.0)	0.795
Comorbidity			
Hypertension (n, %)	9 (19.57)	27 (14.84)	0.432
Cardiovascular disease (n, %)	1 (2.17)	8 (4.40)	0.789
Diabetes (n, %)	5 (10.87)	10 (5.49)	0.327
Chronic liver disease (n, %)	5 (10.87)	7 (3.85)	0.124
Symptoms			
Fever (n, %)	38 (82.61)	135 (74.18)	0.232
Pharyngalgia (n, %)	5 (10.87)	29 (15.93)	0.389
Cough (n, %)	41 (89.13)	146 (80.22)	0.160
Expectoration (n, %)	24 (52.17)	81 (44.51)	0.351
Dyspnea (n, %)	20 (43.48)	60 (32.97)	0.182
Hemoptysis (n, %)	3 (6.52)	4 (2.20)	0.298
Chills (n, %)	5 (10.87)	24 (13.19)	0.673
Myalgia (n, %)	6 (13.04)	18 (9.89)	0.724
Fatigue (n, %)	24 (52.17)	82 (45.05)	0.387
Dizziness (n, %)	3 (6.52)	26 (14.29)	0.158
Headache (n, %)	1 (2.17)	30 (16.48)	**0.011**
Diarrhea (n, %)	11 (23.91)	41 (22.53)	0.841
Nausea (n, %)	2 (4.35)	28 (15.38)	**0.048**
Anorexia (n, %)	26 (56.52)	88 (48.35)	0.322
Vomiting (n, %)	4 (8.70)	21 (11.54)	0.581
Abdominal pain (n, %)	1 (2.17)	6 (3.30)	1.000
Chest CT positive rate (n, %)	46 (100.0)	172 (94.51)	0.221
Chest CT with ground-glass change (n, %)	20 (43.48)	88 (48.35)	0.554
Severe cases (n, %)	17 (36.96)	27 (14.84)	**0.001**
Length of hospital stay, median (IQR), days	13.0 (11.0, 22.5)	16.0 (11.0, 25.0)	0.712
Virus shedding duration, median (IQR), days	17.0 (13.5, 24.0)	18.0 (13.0, 26.0)	0.269
Mortality (n, %)	1 (2.17)	1 (0.55)	0.364

*P* < 0.05 was considered statistically significant (marked in bold)

*COVID-19* Coronavirus disease 2019; *HDL-C* High density lipoprotein cholesterol; *IQR* Inter-quartile range

**Table 4:** Laboratory findings of adult COVID-19 patients with different levels of HDL-C

	Normal range	Low HDL (*n* = 46)	High HDL (*n* = 182)	*P* value
White blood cell count, ×10^9^/L, median (IQR)	4–10	4.78 (3.82, 6.13)	4.58 (3.52, 5.66)	0.627
Lymphocyte count, ×10^9^/L, median (IQR)	0.8–4.0	1.02 (0.72, 1.51)	1.17 (0.84, 1.59)	0.207
Lymphocyte %, median (IQR)	20–40	22.35 (17.58, 30.10)	27.45 (20.05, 33.00)	0.050
Alanine aminotransferase, U/L, median (IQR)	0–42	21.49 (16.13, 30.28)	18.81 (13.81, 26.61)	**0.044**
Aspartate aminotransferase, U/L, median (IQR)	0–37	25.87 (19.53, 34.01)	23.76 (19.25, 28.77)	0.109
Total bilirubin, μmol/L, median (IQR)	3.4–20.5	11.16 (8.16, 15.35)	10.87 (8.68, 16.36)	0.769
C-reactive protein, mg/L, median (IQR)	0–8	27.83 (14.68, 44.74)	12.56 (3.29, 26.88)	**< 0.001**
Erythrocyte sedimentation rate, mm/h, median (IQR)	0–15	52.00 (22.00, 67.75)	39.50 (22.0, 68.25)	0.538
Procalcitonin, ≥0.05 ng/mL, No. (%)	< 0.05	17 (37.0%)	46 (25.3%)	0.113
Creatinine, μmol/L, median (IQR)	21.5–104	53.70 (43.61, 65.02)	50.85 (41.22, 64.02)	0.209
Creatine kinase, U/L, median (IQR)	10–190	83.35 (57.05, 148.63)	69.75 (46.30, 114.45)	0.116
Creatine kinase-MB, U/L, median (IQR)	0–24	9.35 (5.33, 12.50)	9.57 (6.40, 12.92)	0.624

*P* < 0.05 was considered statistically significant (marked in bold)

*COVID-19* Coronavirus disease 2019; *HDL-C* High density lipoprotein cholesterol; *IQR* Inter-quartile range

Considering outcome indicators, patients with low HDL-C showed higher proportion of severe cases (36.96% vs 14.84%, *P* = 0.001) (Table 3). However, there were no significant differences in length of hospitalization, mortality and virus shedding time between the two groups.

**Fig. 1:**
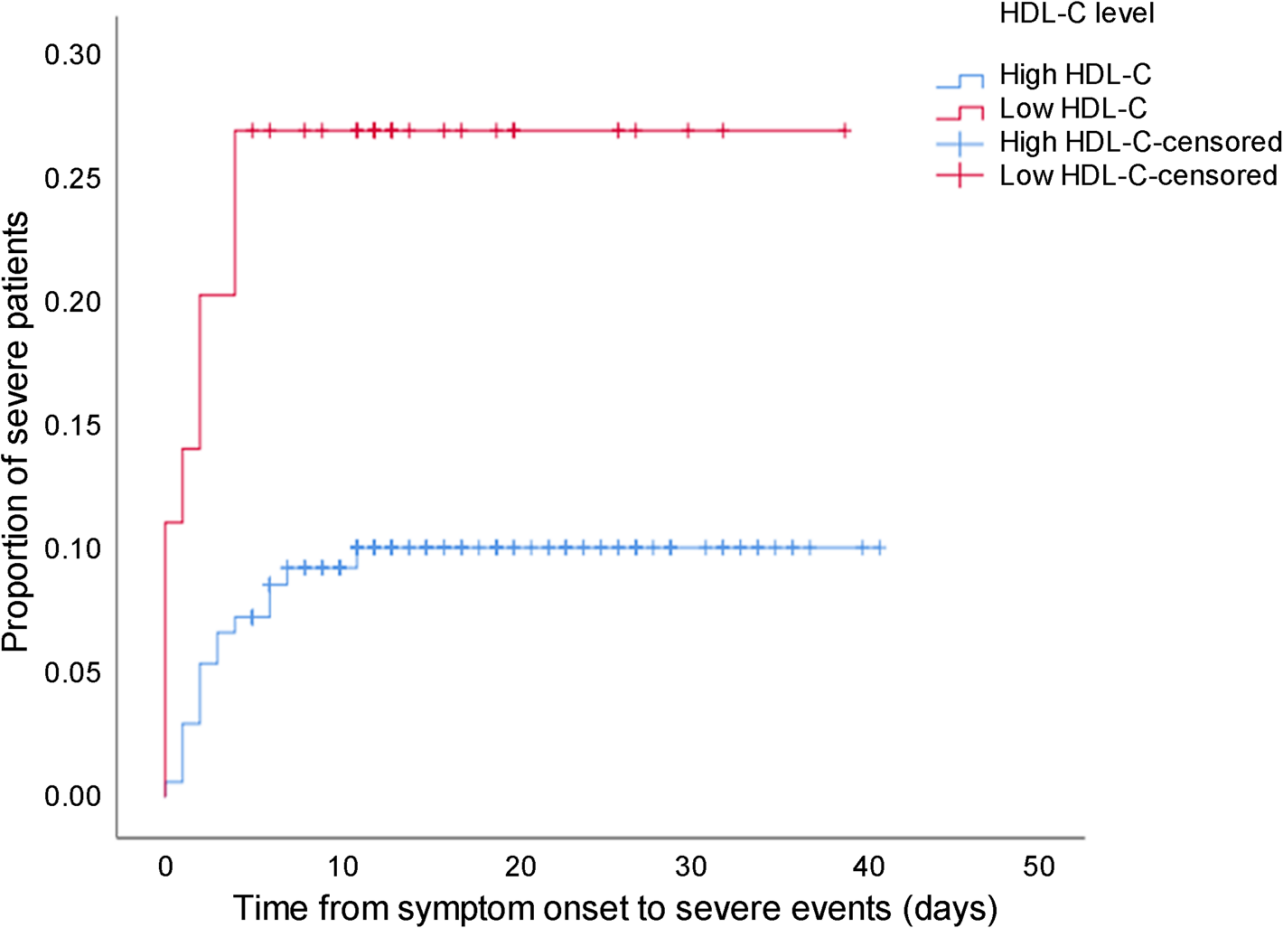
The time-dependent risk of developing severe event in COVID-19 patients with low and high levels of HDL-C using Kaplan-Meier curve. Patients with low HDL-C showed a higher risk of developing severe events compared with those with high HDL-C (Log Rank *P* = 0.009). Abbreviations: COVID-19: coronavirus disease 2019; HDL-C: high-density lipoprotein cholesterol

Moreover, KM curve and Cox regression analysis were employed to analyze the association between HDL-C levels and the risk of developing severe events in non-severe patients after admission. Patients with low HDL-C showed a higher risk of developing severe events compared with those with high HDL-C (Log Rank *P* = 0.009, Fig. 1). After adjusting for age, gender and underlying diseases, patients with low HDL-C still had elevated possibility of developing severe cases than those with high HDL-C (HR 2.827, 95% CI 1.190–6.714, *P* = 0.019) (Table 5).

**Table 5:** Multivariate Cox regression analysis for severe events of adult COVID-19 patients

Variables	HR	95% CI	*P value*
Low HDL-C	2.827	1.190–6.714	**0.019**
Gender	0.621	0.266–1.448	0.270
Age	1.034	1.005–1.064	**0.022**
Hypertension	1.576	0.593–4.188	0.362
Cardiovascular disease	0.888	0.107–7.364	0.912

*P* < 0.05 means statistically significant (marked in bold)

*HR* Adjusted hazard ratios; *COVID-19* Coronavirus disease 2019; *HDL-C* High density lipoprotein cholesterol

## Discussion

This observational study revealed the blood lipids status of adult COVID-19 patients. Through it, it was discovered that low HDL-C was correlated with poor outcomes of adult COVID-19 patients, and provided a basis for HDL-C to predict COVID-19 prognosis, and even became a potential therapeutic target for COVID-19.

In this study, HDL-C levels of adult COVID-19 patients were lower than normal at admission, which was similar to the findings of previous studies [13]. Several studies showed infected patients, especially those with sepsis, always had a significant drop in HDL levels [17, 19, 22, 27–29], but the reason for the decline in HDL level remained unanswered. However, several hypotheses are considered to be possible, including a decrease in HDL synthesis, overconsumption or redistribution of HDL particles from intravascular to the extravascular space [12, 17, 30].

Previous studies also showed that septic patients with low HDL-C level showed higher mortality and other adverse clinical outcomes [22, 29]. Several studies have found significant mortality increase in sepsis patients with an HDL level below 25 mg/dl (0.65 mmol/L) [23, 27], so the clinical characteristics and prognosis of COVID-19 patients with an HDL level above and below 25 mg/dl (0.65 mmol/L) were compared. The study showed that patients with low HDL-C level had higher proportion of severe events, while further regression analysis also revealed that low HDL-C was an independent risk factor for severe events in COVID-19. Hence, HDL-C may serve in a protective role in COVID-19, while COVID-19 patients with reduced HDL-C need proper monitoring and treatment as soon as possible to improve the outcomes.

Excessive inflammation is one of the important features of COVID-19 patients, especially in patients with severe cases or in those who have died [31–34]. It is often manifested by a marked increase in inflammatory factors, such as CRP and interleukins [3, 35]. HDL-C is believed to have an inhibitory effect on inflammation [36–38]. This study showed that patients with low HDL-C had higher level of CRP, which suggested that HDL-C may inhibit the inflammatory response and thus play a protective role in COVID-19 patients. Hence, omega-3 fatty acids may have a potential therapeutic effect in COVID-19, because of its anti-inflammatory properties [39].

The protective effect of HDL-C in bacterial infection is relatively definite. There are many studies that have shown that HDL-C can bind and neutralize the biological toxicity of lipopolysaccharide (LPS) and lipoteichoic acid (LTA) [40–42]. In different experimental septic models, infusion of reconstituted HDL reduced inflammation, decreased bacterial count, attenuated organ injury and improved survival [17, 43, 44], which greatly encouraged the application of HDL in sepsis treatment in the future. However, the role of HDL-C in viral infection remains unclear. In this study, HDL-C was lower in COVID-19 patients, and HDL-C level was negatively correlated with severity of illness, suggesting that HDL-C may be a potential therapeutic target for COVID-19. Some HDL-raising pharmacological compounds have been considered as potential therapies for COVID-19, such as cholesteryl ester transfer protein (CETP)-inhibitors, recombinant cholesterol acyltransferase (LCAT) [40].

### Study strength and limitations

This study found that low HDL-C was correlated with poor outcomes of adult COVID-19 patients, and without any doubt, has its limitations. Firstly, the basic HDL-C data before symptom onset was unknown, so it is uncertain whether the decrease in HDL-C level occurred after infection with SAR-CoV-2. Secondly, previous studies showed that HDLs decrease significantly in the early stage of sepsis, but the time from the symptom onset to the detection of HDLs is different, which may cause some bias in the analysis of the relationship between HDLs and COVID-19. Thirdly, the HDL-C level on the recovery period was undetected, and the correlation between the dynamic changes of HDL-C and the outcome of COVID-19 may be more valuable.

## Conclusion

In summary, this study presented the blood lipid status of COVID-19 patients. Low HDL-C was shown to be associated with higher proportion of severe cases in COVID-19. Moreover, low HDL-C seemed to be an independent risk factor for developing severe events, which suggested that COVID-19 patients with low HDL-C need more intensive treatment and monitoring, and regulating lipoprotein metabolism may be the way forward for COVID-19 treatment in the future.

## Data Availability

The collection of data that supports the findings in this study is available from Public Health Treatment Center of Changsha, but restrictions may apply to the availability of these data, which were used under license for the current study, and so are not publicly available. Data are however available from the authors upon reasonable request and with permission of Public Health Treatment Center of Changsha.
